# Direct observation of unstained biological samples in water using newly developed impedance scanning electron microscopy

**DOI:** 10.1371/journal.pone.0221296

**Published:** 2019-08-20

**Authors:** Toshihiko Ogura

**Affiliations:** Biomedical Research Institute, National Institute of Advanced Industrial Science and Technology (AIST), Central 6, Higashi, Tsukuba, Ibaraki, Japan; VIT University, INDIA

## Abstract

Nanometre-scale observation of specimens in water is indispensable in several scientific fields, such as biology, chemistry, materials science and nanotechnology. Scanning electron microscopy (SEM) obtains high-resolution images of biological samples under high vacuum conditions but requires specific sample-preparation protocols. Observations of unstained biological samples in water require more convenient and less invasive methods. Herein, we have developed a new type of impedance microscopy, namely impedance SEM (IP-SEM), which allows the imaging and sub-micrometer scale examination of various specimens in water. By varying the frequency of the input signal, the proposed system can detect the impedance properties of the sample’s composition at sub-micrometer scale resolution. Besides examining various unstained biological specimens and material samples in water. Furthermore, the proposed system can be used for diverse liquid samples across a broad range of scientific fields, such as nanoparticles, nanotubes and organic and catalytic materials.

## Introduction

Imaging methods with nanometre-scale resolution in liquid or cryogenic conditions are indispensable in various scientific fields, such as biology [1–4], chemistry [5] and nanotechnology [6]. To analyse their functions, biological and organic specimens must be prepared in their natural state under aqueous conditions. Biological specimens are usually analysed by transmission electron microscopy (TEM) and scanning electron microscopy (SEM) [7–10]. However, TEM and SEM observations under high vacuum conditions require specific sample-preparation protocols involving glutaraldehyde fixation, negative staining, cryo-techniques and metal coating or labelling to avoid electrical radiation damage [7–10]. Therefore, more convenient methods are required for observing unstained biological samples in water.

Recently, we developed a novel imaging technology named scanning electron-assisted dielectric microscopy (SE-ADM), which visualises intact biological specimens under aqueous conditions with minimal radiation damage [11–13]. Our system also captures high-contrast images of untreated biological specimens. The biological samples are enclosed in a liquid holder composed of a tungsten (W)-coated silicon nitride (SiN) film, and are not directly exposed to the electron beam (EB). The scanning-EB irradiation is modulated by a beam-blanking unit powered by a function generator at 30–60 kHz [11]. The SE-ADM system enables high-contrast observations while minimising the radiation damage [12, 13] but the frequency of the observing signals cannot exceed 60 kHz [11]. The narrow frequency band of the EB modulation restricts the compositional information of the biological specimens in the frequency spectrum of SE-ADM.

**Fig 1:**
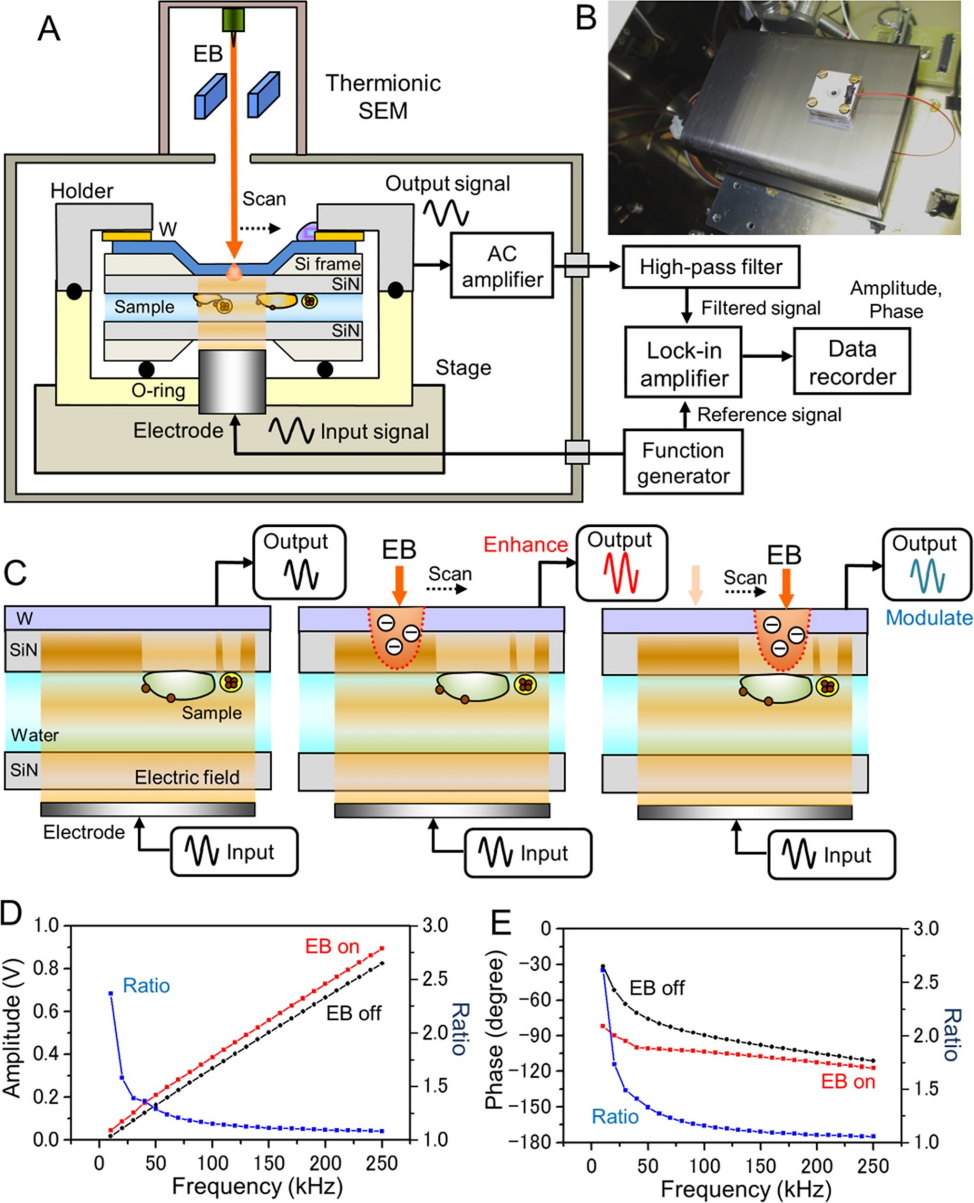
Experimental setup and schematic of the IP-SEM detection mechanism. (A) Schematic of the IP-SEM system. The two SiN films enclosing the liquid sample are sealed by two sample-holding parts. The electrode under the sample holder applies the input voltage signal. The scanning-EB irradiates the upper side of the W-coated SiN film; the output signals from the W-layer are then detected by the AC current pre-amplifier and lock-in-amplifier. (B) Photograph of the sample holder setup on the AC pre-amplifier box. The sample holder was mounted on the Al stage. To detect the output signal, the upper A1 holder part is connected to the AC current pre-amplifier by a cable. (C) Schematic of the IP-SEM imaging mechanism. The input signal is applied to the bottom electrode, and the output signal from the upper W-layer is detected (left panel). When irradiated by the EB, the W-coated SiN film scatters and absorbs the electrons, modulating the SiN resistance and impedance at the EB position. The resistance modulation enhances the output signal (centre panel). When the EB irradiates the biological specimen, the output signal is attenuated by the specimen’s material (right panel). (D) and (E) Output amplitude (D) and output phase (E) of the IP-SEM system versus input frequency without sample in the holder. The EB irradiation enhances the amplitude and attenuates the phase (EB acceleration voltage 4 kV and EB current 1 nA).

The present study introduces a newly developed impedance-SEM (IP-SEM), which observes untreated biological specimens and 500-nm beads in liquid environments (Fig 1). Wet biological specimens are enclosed within two SiN films, the upper of which is coated with tungsten (W). A sine-wave voltage signal is applied to the bottom electrode (Fig 1A), generating electric-field oscillations that are transmitted through the samples in water to the upper W-coated SiN film. The EB is irradiated on the W-coated SiN film. Finally, the output signal from the W-layer (which contains the structural and compositional information of the samples) is detected by the lock-in-amplifier (Fig 1). Our system offers clear, sub-mircrometer resolution imaging and compositional analysis of biological and organic materials in aqueous conditions.

## Materials and methods

### Tungsten deposition condition on the upper SiN film using the sputtering device

A 50 nm-thick SiN film supported by a 0.4 × 0.4 mm^2^ window in a Si frame (area 4 × 4 mm^2^, thickness 0.381 mm, Silson Ltd., UK) was coated with tungsten using a magnetron sputtering device (Model MSP-30T, Vacuum Device Inc., Japan). Tungsten was sprayed for 10 s under 1.0 Pa argon pressure and a current of 200 mA, producing a 10 nm-thick coating. The distance between the sputter target and the SiN film was 50 mm.

### Sample preparation

Poly-lactic-acid (PLA) particles (diameter, 500 nm; density, 1.0 g/mL) in water were obtained from Micromod Partikeltechnologie GmbH (Rostock, Germany). The beads in their liquid solution (1 μL) were added to the liquid-sample holder as a control sample, which was then sealed. The whole-milk specimen was then prepared from the ‘Meijioishii-gyunyu’ commercial product, obtained in a 200-mL bottle pack from Meiji Co., Ltd. (Tokyo, Japan). The sample solution (1 μL) was introduced to the liquid-sample holder.

### The liquid-sample holder and stage

The newly developed sample holder maintained the sample solution at near-atmospheric pressure in the space between a W-coated SiN film and an uncoated film (both films were 50 nm-thick). The two SiN films incorporating the liquid sample were sealed in two sample-holding parts with an O-ring, and the holding pieces were sealed by four screws. The sample holder comprised an upper aluminium part and a lower acrylic resin part. The upper part was connected to a pre-amplifier, allowing conduction to the metal layer on the SiN film (Fig 1A and 1B). A voltage signal was applied to the electrode installed at the centre of the acrylic part. The frequency of the voltage signal was varied from 20 to 250 kHz.

### IP-SEM system and setup

The IP-SEM imaging system based on thermionic-SEM (JSM-6390, JEOL, Japan) is shown in Fig 1A. The voltage signal input to the electrode in the acrylic holder was applied by a function generator (WF1948, NF Co., Japan). The signals were sine waves of varying frequency (10–250 kHz) and fixed amplitude (0.1–20 V peak to peak). The liquid-sample holder was mounted onto the SEM stage, and the aluminium holder mounted with the W-coated SiN film was connected to the AC current pre-amplifier of a trans-impedance amplifier (510 kΩ, 1.02 × 10^6^ gain) under the holder (Fig 1B). The input signal from the bottom electrode was transmitted through the liquid sample to the W-coated SiN film at the upper side (Fig 1C, left panel). The EB was irradiated on and almost absorbed by the W-layer on the upper SiN film, thereby slightly shielding the sample from radiation damage (Fig 1C, centre panel). The frequency signal output from the AC current pre-amplifier was passed through a high-pass filter (HPF, cut-off frequency = 10 kHz) and fed into the lock-in amplifier (LI5650, NF Co., Japan). The HPF signal, lock-in amplifier output and X–Y scan signal were logged by a data recorder (EZ7510, NF Co., Japan) at a sampling frequency of 50 kHz. The SEM images (1,280 × 960 pixels) were captured at 150–10,000× magnification. The scanning time and working distance were 80–120 s and 7–8 mm, respectively. The EB acceleration voltage was 3–7 kV, and the operating current was 37–1,000 pA.

### Image processing

The IP-SEM signal data of amplitude and phase from the data recorder were transferred to a personal computer (Intel Core i7, 3.2 GHz, Windows 7), and the IP-SEM images were processed by the image processing toolbox in Matlab R2016b (Math Works Inc., USA). The original IP-SEM images were filtered through a two-dimensional Gaussian filter (GF) with a kernel size of (7 × 7) pixels and a radius of 1.2σ. The background was removed by subtracting the IP-SEM images from the filtered images using a broad GF (kernel size 201 × 201 pixels, radius 100σ). Finally, amplitude and phase images were changed to 8-bit grey scale format.

Peak signal to noise ratio (PSNR) is very popular quality check indicator of images [14]. *PSNR* was calculated by Eq (1).

$$PSNR=10\;\log_{10}\frac{I^{2}}{MSE}$$ (1)

Where *MSE* is mean squared error of without sample region, *I* is the maximum pixel intensity of sample region (e.g. 255 for 8-bit grey scale image).

### Calculation of impedance value from observation current output

The lock-in-amplifier of IP-SEM system outputs the voltage values without offset at EB-off condition of *X* and *Y*, which is called the 'in-phase' and 'qunadrature' components. *X* and *Y* observed voltage values of mV range. The output current values of *I*_*X*_ and *I*_*Y*_ are calculated by the gain of current pre-amplifier (*G* = 1.02 × 10^6^) as follows:

$$I_{X}=\frac{X}{G}$$ (2)

$$I_{Y}=\frac{Y}{G}$$ (3)

The *I*_*X*_ and *I*_*Y*_ are translated into magnitude (*I*_*R*_) and phase (*I*_*θ*_) as follows:

$$I_{R}=\sqrt{{I_{X}}^{2}+{I_{Y}}^{2}}$$ (4)

$$I_{\theta }=\tan^{-1}\frac{I_{Y}}{I_{X}}$$ (5)

The impedance values *Z*_*f*_ is calculated by input voltage *V*_*f*_ and *I*_*X*_, *I*_*Y*_ as follows:

$$\begin{array}{l} Z_{f}=\frac{V_{f}}{I_{X}+i\;I_{Y}}\\ \;=\frac{V_{f}I_{X}}{{I_{X}}^{2}+{I_{Y}}^{2}}-i\frac{V_{f}I_{Y}}{{I_{X}}^{2}+{I_{Y}}^{2}} \end{array}$$ (6)

Where *i* is the imaginary unit (*i*^2^ = -1). Impedance *Z*_*f*_ is calculated by each input voltage *V*_*f*_ between 20–250 kHz frequency. *Z*_*f*_ is constructed by resistance of real part (*Z*_*R*_) and reactance of imaginary part (*Z*_*X*_). The magnitude (|Z|) and phase (θ) of the impedance are calculated by the following equations:

$$|Z|=\sqrt{{Z_{R}}^{2}+{Z_{X}}^{2}}$$ (7)

$$\theta =\tan^{-1}\frac{Z_{X}}{Z_{R}}$$ (8)

## Results

Fig 1 outlines our IP-SEM system based on thermionic SEM. Intact liquid specimens were introduced into the space between the two SiN films. Because the irradiated electrons were absorbed in the W-layer of the upper film, the insulation resistance of the film was reduced in the region of the EB position (with an approximate EB spot diameter) [11]. The reduced insulation resistance enhanced the impedance signal from the W-layer at this position (Fig 1C, centre panel). When the EB was irradiated on the water region of the sample holder, we detected the water background signal between the bottom electrode and the EB-irradiated position in the upper SiN film. When the EB was scanned over the sample region, the output signal (including the impedance information) was modulated by the specimen compositions (Fig 1C, right panel). The sample impedance information was constructed into an IP-SEM image by the lock-in-amplifier output and EB scan signals. Panels D and E of Fig 1 plot the amplitudes and phases of the output signal from a lock-in-amplifier, respectively, as functions of frequency without the specimen in the sample holder, and with the EB irradiation turned on or off. The EB irradiation on the W-coated SiN film enhanced the output amplitude of the lock-in-amplifier. The input-to-output ratio gradually reduced with increasing input frequency (Fig 1D). The phase and the EB on–off ratio of the output signal similarly reduced at higher input frequencies (Fig 1E). Clearly, the EB irradiation enhanced and modulated the amplitude and phase of the output signal.

**Fig 2:**
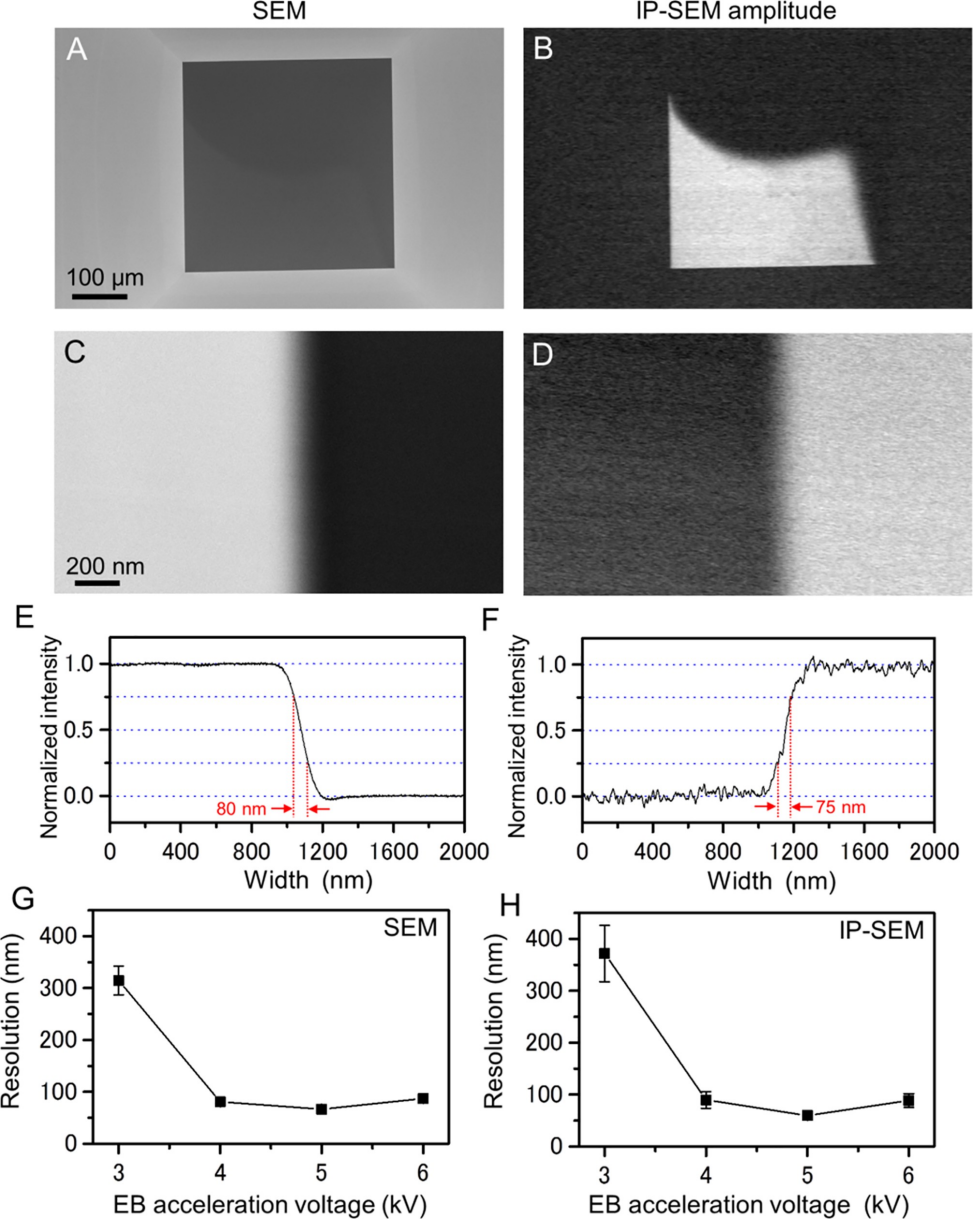
Comparison of spatial resolution between SEM and IP-SEM. (A) A SEM image of the pure water in the atmospheric holder by the secondary electron signal. (B) Simultaneous observation of a current amplitude image by the IP-SEM system (EB accelerating voltage 4 kV, EB current 140 pA, input frequency 50 kHz, magnification 150×). (C) A high-magnification image of the SiN film edge by SEM, scanned at 60,000×. (D) Simultaneous observation of an amplitude image by the IP-SEM system. (E) Averaged lineout across the SiN film edge in (C). Spatial resolution of the thermionic SEM is 80 nm under same EB condition of (A). That is defined as the distance over which the intensity decreases from 0.75 to 0.25 in the lineout. (F) The spatial resolution of the IP-SEM system was 75 nm. (G) Spatial resolution of SEM image across the SiN film edge under 3–6 kV EB acceleration voltage, each EB voltage resolution is averaged of five different scanned areas of the SiN film edges. The EB currents of 3–6 kV acceleration voltages are 820 pA, 140 pA, 40 pA and 37 pA. (H) Spatial resolution of IP-SEM system. The resolution of SEM and IP-SEM system is almost same values. Scale bars: 100 μm in (A), 200 nm in (C).

Initially, we calculated the spatial resolution of our IP-SEM system by the edge of SiN film image with pure-water within the holder (Fig 2). In the low-magnification, the SiN window is clearly observed in both images of SEM and the current amplitude images of IP-SEM (Fig 2A and 2B). To measure spatial resolution, the SiN edge was scanned at 60,000× magnification (Fig 2C and 2D). The averaged lineout across the SiN edge is shown in Fig 2E and 2F. The spatial resolutions of SEM and IP-SEM are 80 nm and 75 nm, that is defined as the distance over which the normalized intensity decreases from 0.75 to 0.25 in the lineout. The IP-SEM resolutions of 3–6 kV EB accelerations were almost same as secondary electron images of SEM (Fig 2G and 2H). These results were strongly suggested that the IP-SEM resolution is influenced by the EB spot diameter.

**Fig 3:**
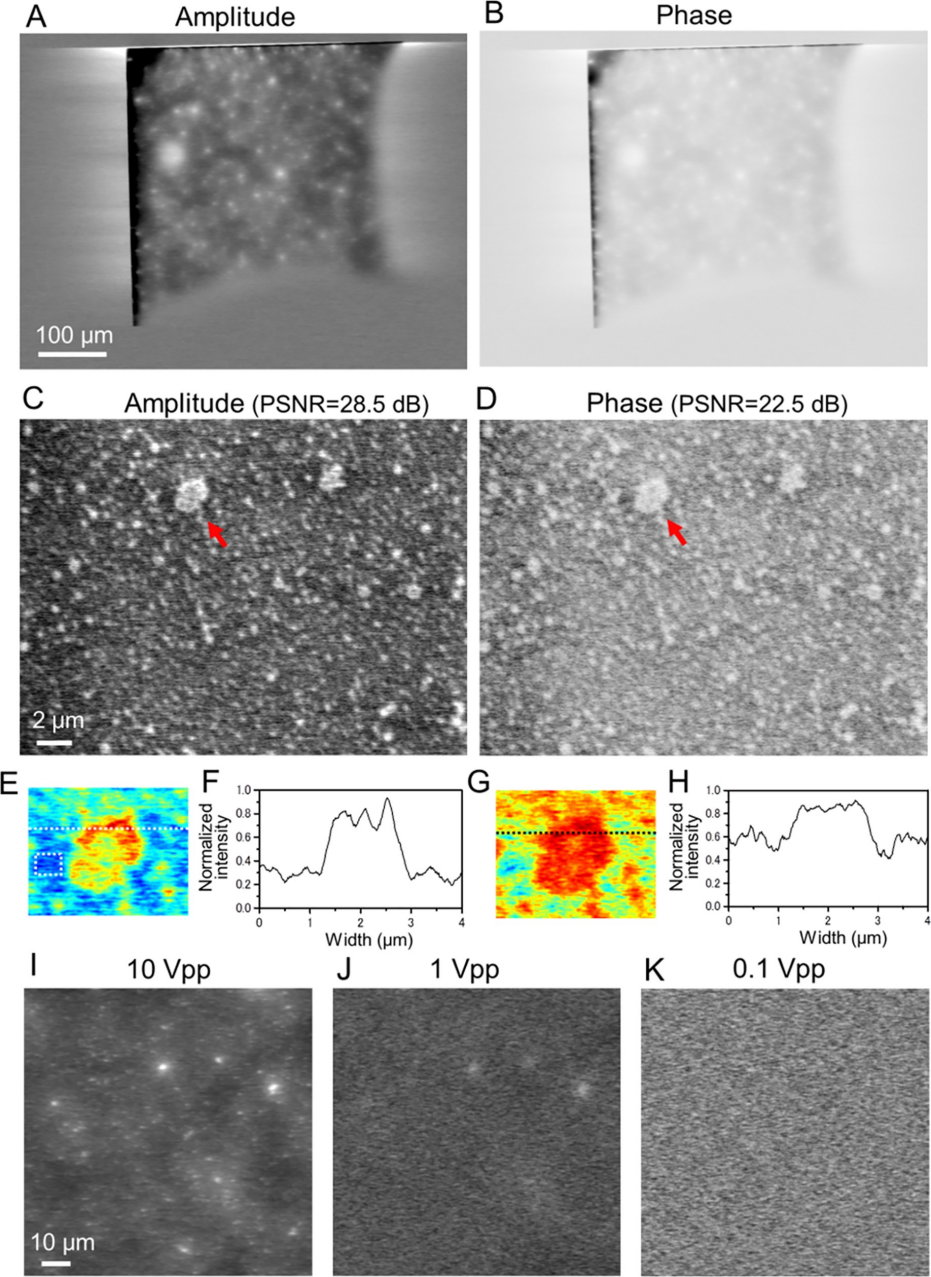
Amplitude and phase images of PLA beads in water observed by the IP-SEM system. (A) and (B): Amplitude (A) and phase (B) images of 500 nm-diameter PLA beads in water, simultaneously obtained from the lock-in amplifier (EB acceleration voltage 7 kV, EB current 300 pA, input frequency 50 kHz, magnification 200×). The large white particles in (A) are bead aggregates. (C) Amplitude image of the PLA beads in water at high magnification (5,000×). The white particles (500 nm-diameter beads) are dispersed over the whole area. PSNR of this image is 28.5 dB, which is calculated by the beads aggregation area and no-beads area using Eq (1). (D) Simultaneously obtained phase image. PSNR is 22.5 dB. (E) An expanded pseudo-colour map of the aggregation beads indicating a red arrow in (C). The noise level of MSE was measured at the no-beads area in the white square. (F) Line plots of the aggregation beads along the white dotted lines in (E). (G) An expanded pseudo-colour map of the aggregation beads indicating a red arrow in (D). (H) Line plots of the black dotted lines in (G). (I) Amplitude image of the beads at 10 V_pp_ sine wave input, (magnification 500×, input frequency 50 kHz, EB accelerating voltage 7 kV). The white beads are clearly observed. (J) Amplitude image of the beads at 1 V_pp_. The contrast of the white beads is decreased. (K) Amplitude image of the beads at 0.1 V_pp_. The white beads have completely disappeared. These amplitude images were reversed contrast. Scale bars: 100 μm in (A), 2 μm in (C) and 10 μm in (I).

Next, we observed IP-SEM images of the 500 nm-diameter PLA beads in water at an input frequency of 50 kHz (Fig 3). In the low-magnification (200×) amplitude and phase images, large particles of white contrast are dispersed over the whole area of the SiN film window (Fig 3A and 3B). These particles are aggregates of the 500 nm-diameter particles. The beads in water were further analysed at high magnification (5,000×; see Fig 3C and 3D). Again, the IP-SEM amplitude image shows clear white-contrasted particles over the whole area, including a few aggregates of diameter 1–2 μm (pointed by a red arrow in Fig 3C). The image quality value of PSNR of this image is 28.5 dB. The contrast in the phase image of PSNR (22.5 dB) is lower than in the amplitude image (Fig 3D). To investigate the aggregation beads in detail, we created a pseudo-colour map and its line-plots of a beads aggregation area (Fig 3E–3H), it is also suggested that the amplitude current image is higher quality than phase image. To ascertain whether the IP-SEM images of amplitude and phase IP-SEM images are constructed from the frequency signal input to the electrode, we observed the same sample after sequentially decrementing the amplitude of the input signal (Fig 3I–3K). The contrast in the IP-SEM amplitude image gradually disappeared as the input signal amplitude to the electrode decreased from 10 to 0.1 V_pp_, despite constant EB conditions (Fig 3I–3K). Therefore, the amplitude and phase images of the IP-SEM system are constructed not from the scanning-EB signals, but from the signal input to the electrode. The scanning-EB functions only as the position indicator of the detection area on the W-coated SiN film.

**Fig 4:**
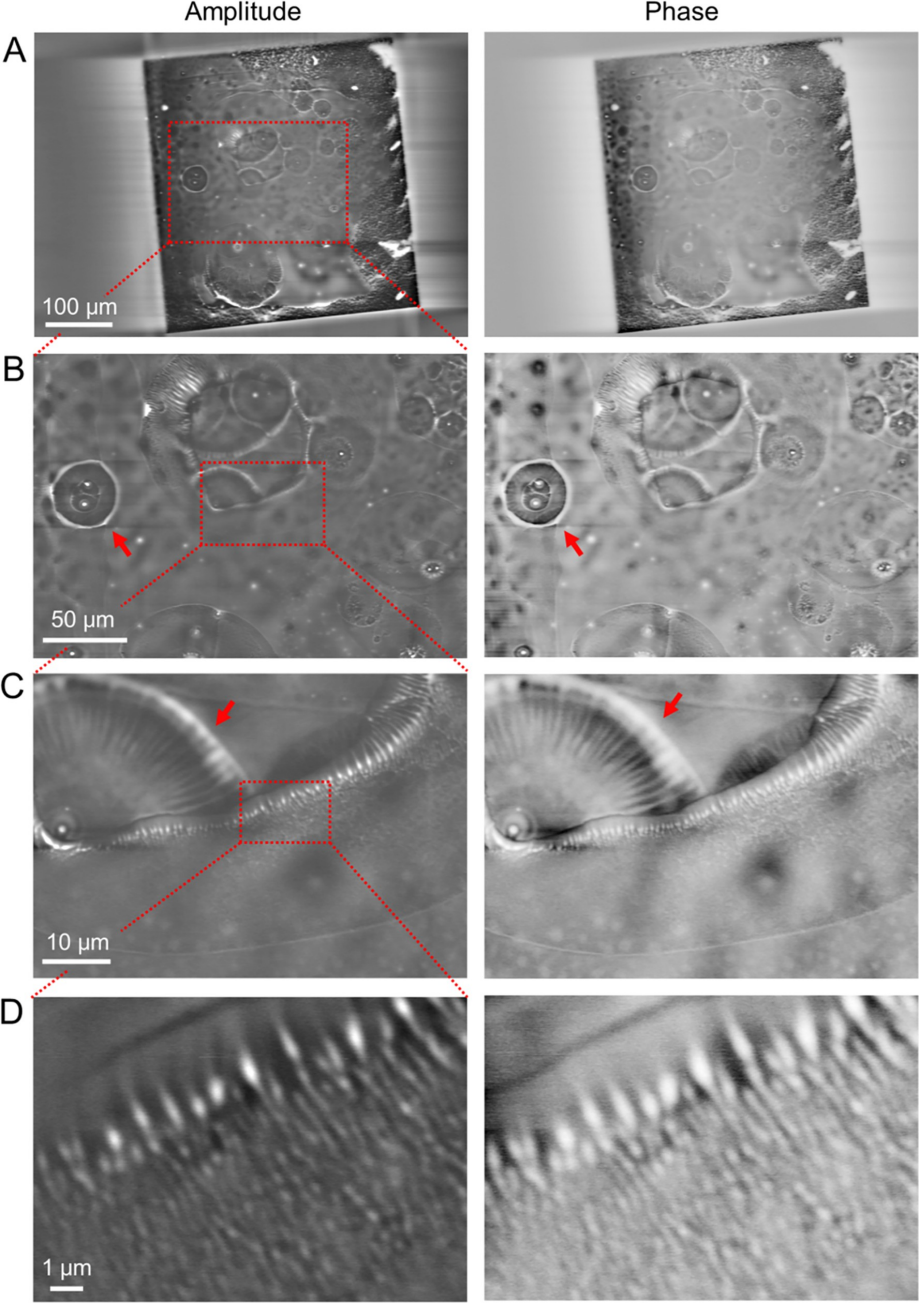
Amplitude and phase images of PLA beads in the bubble area of a wet environment observed by the IP-SEM system. (A) Amplitude and phase images of the 500 nm-diameter PLA beads in water with bubbles (EB accelerating voltage 5 kV, EB current 400 pA, input frequency 50 kHz, magnification 200×). Large spherical bubble areas are clarified in both images. (B) Expanded amplitude and phase images of the red boxed area in (A) at 500× magnification. Both images clarify the white contrast of aggregation beads and the spherical bubble areas. (C) Amplitude and phase images of the air–water boundary of the bubble in the red boxed area of (B) at high-magnification (2,000×). (D) High-magnification (10,000×) amplitude and phase images of the air–water boundary of the bubble. Both images in (C) and (D) clarify the white-contrasted beads along the bubble boundary. These amplitude images were reversed contrast. Scale bars: 100 μm in (A), 50 μm in (B), 10 μm in (C) and 1 μm in (D).

Observing the drying conditions of the nanoparticles in liquid sample is important in the production and quality control of materials. To analyse drying process in the beads particle in vapor, we observed the bubble area of the 500-nm PLA beads in water using the IP-SEM system (Fig 4). In the low-magnification amplitude and phase images of a 50-kHz input signal, large circular bubbles of diameter 10–50 μm were visible (Fig 4A). At 500× magnification, the air–water boundary of the bubble exhibited ring-like structures of white contrast (Fig 4B, red arrows). To analyse the details, we observed the peripheral region of the bubble area at high magnification (Fig 4C and 4D). Fig 4C shows the air–bubble boundary of the bubble at 2,000× magnification. Interestingly, the white-contrasted beads are arranged along the bubble boundary. A radial structure appears at the left side of the image (Fig 4C, red arrows). The radial pattern, which is better clarified in the phase contrast image than in the amplitude image, is probably constructed by self-organisation of the water evaporation in the bubble. In the high-magnification image of the bubble edge, the white-contrasted beads are aligned along the boundary in the water region of the bubble (Fig 4D). Along the bottom side of Fig 4D, the beads are randomly distributed within the water region. The beads’ contrast slightly differs between the amplitude and phase images (Fig 4D), being lighter in the phase image.

**Fig 5:**
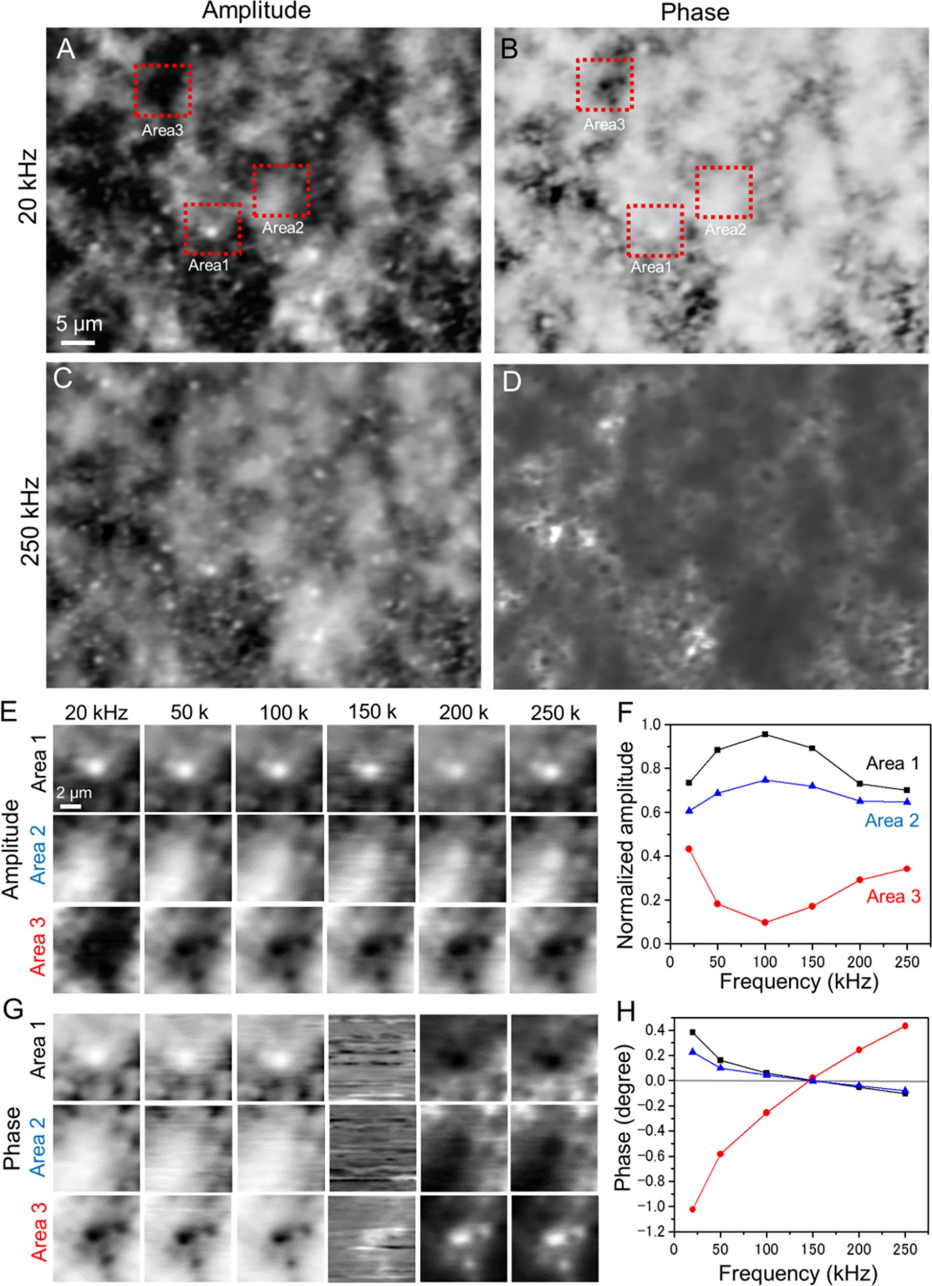
Amplitude and phase images of whole-milk sample in liquid conditions observed by the IP-SEM system. (A) Amplitude image of the whole-milk sample (input frequency 20 kHz, EB accelerating voltage 7 kV, EB current 500 pA, magnification 1,000×). Black and white particles appear within a white cloud-like structure. (B) Simultaneously obtained phase image. (C) Amplitude image of the area scanned in (A) with a 250-kHz input signal (other conditions unchanged). These amplitude images were reversed contrast. (D) Simultaneously obtained phase image. (E) Amplitude images of the white and black particles in Areas 1–3 in (A) obtained at various input frequencies of 20–250 kHz. The white particle is insensitive to input frequency in Area-1, but fades with increasing frequency in Area-2. (F) Normalised amplitude versus input frequency for the white and black particles in the centres of Areas 1–3 in (E). (G) Phase images of the white and black particles in Areas 1–3 in (B) obtained at various input frequencies. The phase contrast reverses above 150 kHz. (H) Phase versus input frequency for the white and black particles in the centres of Areas 1–3 in (G). The phase lines cross the 0° line at 150 kHz. Scale bars: 5 μm in (A) and 2 μm in (E).

To analyse the biological sample, we observed the IP-SEM amplitude and phase images of the whole-milk specimen under liquid conditions, varying the input frequency from 20 to 250 kHz (Fig 5). Whole milk mainly comprises milk-fat globules and casein micelles [15–17]. The IP-SEM image of the whole-milk sample taken at 20 kHz shows black and white particles dispersed in a white cloud-like structure (Fig 5A and 5B). The white contrasts of the particles and cloud-like structure are enhanced in the phase image (Fig 5B). At 250 kHz, the white contrast of the cloud-like structure decreased in the amplitude image while the particle contrast increased (Fig 5C). The contrast in the phase image taken at 250 kHz (Fig 5D) is reversed from that at 20 kHz (Fig 5B).

In Area-1 of Fig 5A, the particles in the amplitude image show similar contrast and structure at all input frequencies (20–250 kHz; see upper panels of Fig 5E). Conversely, the contrast of the white particles in Area-2 increased with increasing input frequency (Fig 5E, middle panels). Area-3 contained black particles, which were broad and large in the amplitude image of the 20-kHz input, but resolved into four small black particles at inputs of 50 kHz and higher (Fig 5E, bottom panels). The normalised amplitudes of the white particles increased as the input signal increased from 20 kHz to 100 kHz, and decreased at frequencies above 150 kHz (Fig 5F, Areas 1 and 2). By contrast, the normalised amplitude of the black particles in Area-3 decreased between 20 and 100 kHz and increased at frequencies above 150 kHz (Fig 5F, Area-3). In the phase images, the contrasts of both the black and white particles were consistent at input frequencies up to 150 kHz and then reversed (Fig 5G). The phases of the white particles gradually decreased with increasing input frequency in Areas 1 and 2, but rapidly increased with frequency in Area-3 (Fig 5H). The contrast in the phase images of current output reversed at 150 kHz because all phase lines crossed over the 0-degree line at this frequency. Fig 5H and Fig 1E show the different frequency characteristics. Due to the high-dielectric values of water, this difference is caused by the sample holder with or without liquid sample.

**Fig 6:**
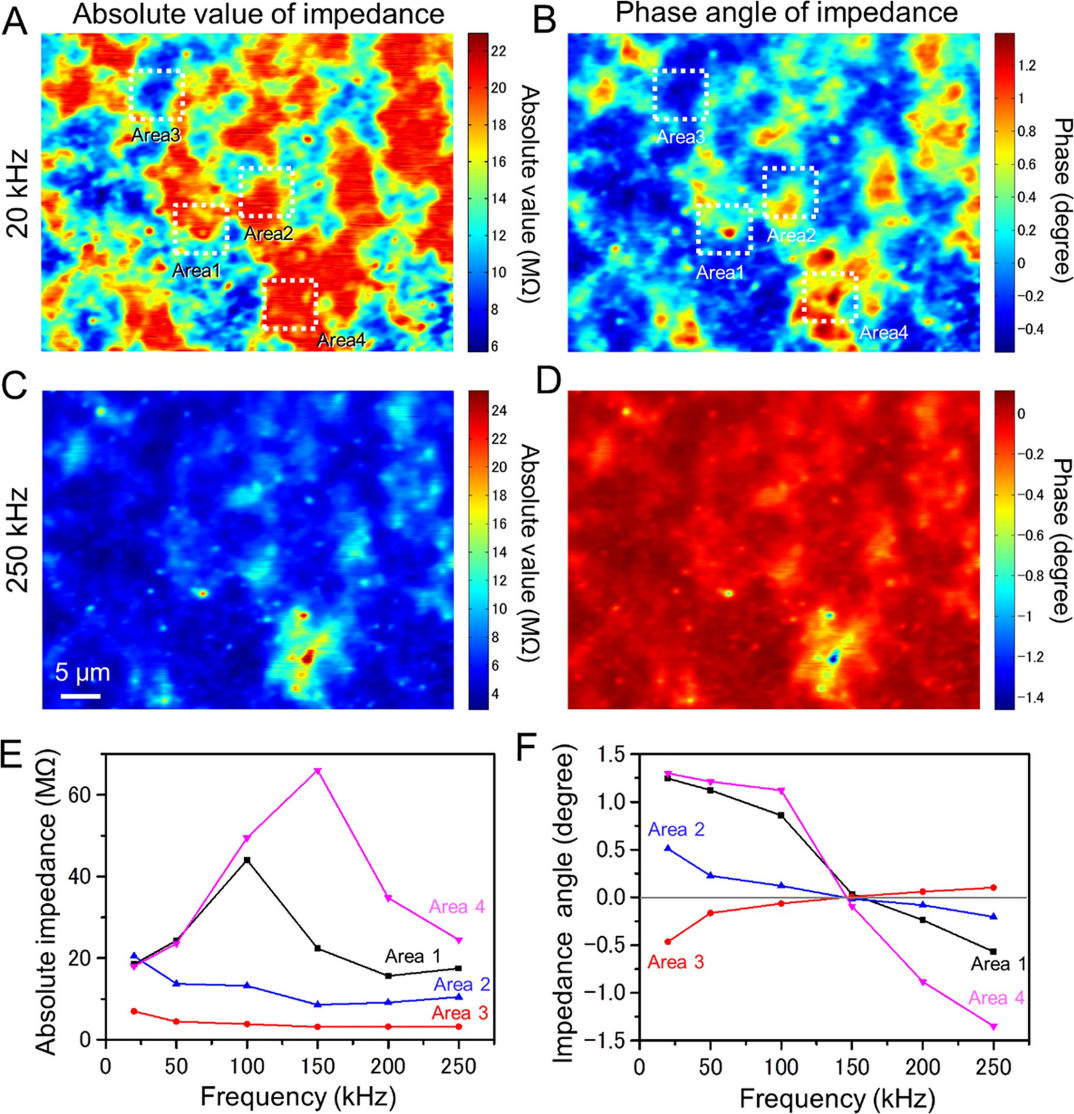
The impedance amplitude and phase colour maps of the whole milk sample. (A) A colour map of absolute impedance of the whole milk sample (EB accelerating voltage 7 kV, EB current 400 pA, input frequency 20 kHz, magnification 1,000×). The impedance range in map is 6–22 MΩ. (B) Simultaneously obtained an impedance phase map. (C) Amplitude map of the same area scanned in (A) with a 250-kHz input signal (other conditions unchanged). The impedance range of its map is expanding to 4–25 MΩ. (D) Simultaneously obtained an impedance phase map. (E) The absolute impedance versus input frequency of the white and black particles in the centres of Areas 1–4 in (A). Areas 1 and 4 shows sharp peak at 100 kHz and 150 kHz input frequency. By contrast, the amplitude of Area-2 shows almost flatten in whole frequency. (F) A frequency characteristic of phase at the white and black particles in the centres of Areas 1–4. Scale bar, 5 μm in (C).

Finally, we calculated impedance amplitude and its phase images using the whole-milk sample (Fig 6). Impedance is defined as the ratio of AC voltage to the current. Our IP-SEM system detects the current output signal using a lock-in-amplifier through an AC current pre-amplifier. Therefore, the impedance amplitude and phase values are calculated by the gain of preamplifier and input voltage (see Materials and Methods section). The colour maps of absolute impedance and phase angle images show Fig 6A–6D. In 20 kHz input frequency, the absolute value and phase angle images of impedance show patchy pattern in whole area (Fig 6A and 6B). In 250 kHz input frequency, the clear sharp peaks in bottom area appear in both images of amplitude and phase (Fig 6C and 6D).

The frequency characteristic of absolute impedance value of Area 1–4 shows in Fig 6E. The absolute impedance of the Area-1 is decreased after reaching a peak at gradually increasing 100 kHz from 20 kHz. Impedance of the Area-4 is peaked at 150 kHz and 66 MΩ, which is increased to three times from 20 kHz of 18 MΩ. Areas 2 and 3 are constant or decreased slightly with an increase in input frequency. The phase impedance image of Areas 1 and 4 gradually decreases with increasing 20–100 kHz frequency, which shows sharp decrease in 100 kHz or more. The difference in characteristics are caused by the difference of the reactance component of the impedance due to the compositions of the sample, which is imaginary part of impedance. Therefore, in the IP-SEM system, it is possible to observe the difference in the sample composition by analysing the frequency characteristics of impedance.

## Discussion

In this study, we introduced our newly developed IP-SEM system placed in a thermionic-SEM chamber (Fig 1A and 1B). Our IP-SEM imaging system provided high-contrast images of PLA 500-nm beads and whole-milk specimens in aqueous conditions (Figs 3–6). The untreated specimens in water are introduced into the space between two SiN films, and the input generating the image is a voltage signal applied to the electrode in the acrylic holder (Fig 1A). The input signal is conveyed through the sample to the W-coated SiN film at the upper side (Fig 1C). When the upper film is irradiated with a scanning EB, the changes in the sample impedance information are detected in the output current signal from the W-layer (Fig 1C). Our new IP-SEM system simultaneously captures the amplitude and phase images of untreated samples in aqueous conditions (Figs 3–6). It can also detect the sample compositions from amplitude and phase of impedance information at varying input frequencies of the multi-scanning EBs (Figs 5 and 6). The phase angle images reversed contrast at 150 kHz frequency, because of the frequency characteristics cross over 0 degrees lines in the frequency (Figs 5F and 6F). This characteristic of phase shift is caused by differences in component of sample reactance, that is imaginary part of the impedance. We assume that the minimum phase point of 150 kHz is the resonance frequency of the sample. The resonance frequency is minimized phase angle, because of equivalent values of the inductance and capacitance. The absolute value of impedance is maximizing at the minimizing phase of 150-kHz frequency, it is suggested that the equivalent electric circuit of sample is the parallel RLC circuit. Therefore, near the resonant frequency, we detect important information as resistance and reactance of the equivalent electric circuit of the sample. The input frequency range is preferable to set at including a resonant frequency of samples.

In our IP-SEM system, the W-coated SiN film is irradiated to the high-current EB of 37–1000 pA. Therefore, the contamination is deposited on the W-coated SiN film. To avoid the contamination deposit, the EB current has to decrease under 10 pA level. However, low-current EB is decreasing the output signal and SNR. In addition, our IP-SEM images slightly exhibit the horizontal line noise (Figs 3–6). This noise is caused by the low-frequency noise of the pre-amplifier and the sample shift or movement in holder. Therefore, we will try to development of the low-noise and wide range pre-amplifier and the high-stability sample holder at the next generation IP-SEM.

To observe mammalian cultured cell, introducing the temperature control stage in IP-SEM system will be effective. At long time observation of mammalian cells, it is recommended that the holder temperature maintains 37°C. The temperature control stage to keep 37°C will be helpful to the healthy state maintenance of the mammalian cells in the holder.

The thickness of the W-layer on SiN film gives a slight effect on the image quality (S1 Fig). At the W-thickness of 5, 10 and 20 nm, the current amplitude images of the SiN windows under air condition show clear contrast (S1A–S1C Fig). The line plots of the SiN film centre clearly exhibit the difference between the SiN film signal and the outside frame (S1D–S1F Fig). Output current decreases as the W-layer is increased (S1G Fig). However, PSNR of quality value of the image is almost constant with respect to the thickness of the W-layer (S1H Fig).

Furthermore, we analysed the electron trajectories on the W-coated SiN film with water using the Monte Carlo (MC) simulation in CASINO ver. 2.42 [18]. We estimated that the density and thickness of the W layer and SiN film were 19.3 g/cm^3^ and 10 nm, 3.12 g/cm^3^ and 50 nm, respectively. The simulation parameters were set as follows: 1,000,000 electrons, 3–7 kV acceleration voltage, and 30 nm EB diameter. As a result, scattering range of the EB spreads by increasing the accelerating voltage (S2A and S2B Fig). Moreover, the trajectory of the irradiated electrons was found to reach a depth of approximately 1 μm in water at 7 kV EB acceleration (S2C Fig). However, transmission range is gradually decreased by lowering the accelerating voltage, the 4 kV acceleration EB is lower than 200 nm penetration area. Therefore, at 3–4 kV EB acceleration condition, we enable to observe the impedance image at low-radiation damage. However, in the acceleration voltage 5 kV or higher, the sample in holder is affected to the penetrated electrons from the W-coated SiN film.

The dielectric properties of animal cells at various input frequencies (10 kHz–10 MHz) were measured in a previous study [19]. The dielectric-properties method analyses the frequency spectrum of the dielectric behaviour of bulk biological specimens in water using an impedance analyser [19, 20]. Besides capturing the dielectric properties, our IP-SEM system observes the biological samples at nanometre resolution, which is an important advantage of our system. The input frequency of the present IP-SEM system cannot exceed 250 kHz, the operating limit of the lock-in-amplifier. In the next IP-SEM system, we plan to use a high-speed lock-in-amplifier, enabling observations at 10 MHz and higher. Other groups have reported imaging systems using electrical impedance tomography (EIT) [21–23], which are accurate to a few cm. The EIT method was applied to three-dimensional (3D) imaging of the human body and head using an electrode array system [22–24]. Our system can be easily adapted to a similar 3D imaging system with a multi-electrode array.

The spatial resolution of an IP-SEM image is influenced by the EB spot diameter (Fig 2) and the thickness of the W-coated SiN film (S1 Fig). In our IP-SEM system, the spatial resolution is 60–380 nm, which is influenced by EB acceleration voltage and current (Fig 2H). Interestingly, the IP-SEM spatial resolution is almost same as SEM resolution by the secondary electron image. It is strongly suggested that the IP-SEM resolution is related to EB spot diameter. The spatial resolution of current IP-SEM system is lower than other liquid imaging methods e.g. atomic force microscopy (AFM) and SE-ADM [11–13, 25]. AFM observes high-resolution image of surface on various samples in water [25]. However, this system is hard to detect inner structural information in the sample. By contrast, our IP-SEM system enables observation of the inner structure of samples. Recently, we had been developing the dielectric microscopy of SE-ADM system [11–13]. This system allows for high-resolution imaging of various biological samples in liquid condition at the spatial resolution of 8-nm [11–13]. In the present IP-SEM system, the spatial resolution is 60–380 nm at the EB acceleration of 3–6 kV (Fig 2H), which is lower than SE-ADM system. In advantage of IP-SEM, our system is enabled to analyse the frequency characteristics from the impedance image in the sample. Moreover, in the next-generation IP-SEM system, the spatial resolution will be improved by adopting high-resolution field-emission SEM of 3-nm EB spot diameter and reducing the thickness of the SiN film in the sample holder to below 50 nm. Some biotech companies have recently reduced the SiN film thickness to 10 nm, which is probably beneficial for improving the spatial resolution. We expect that our next high-resolution IP-SEM system, enabling 3D structural analysis using a linear array electrode system.

## Conclusion

We newly developed an impedance microscopy (an IP-SEM system) based on thermionic SEM. This system imaged PLA beads and intact milk samples in water at high contrast. For varying frequency inputs, it also detected the dielectric properties of the sample compositions at sub-micrometer scale resolution. Our new method is suitable for analysing various unfixed and unstained biological specimens in water environments. Furthermore, our IP-SEM system can observe diverse liquid samples across a broad range of scientific fields, such as nanoparticles, nanotubes and organic and catalytic materials.

## Supporting information

S1 Fig The amplitude current images of the W-coated SiN films under various W thickness. (A)–(C) The current amplitude images of 5–20 nm W-coated SiN film at the atmospheric condition in the holder. (D)–(F) The horizontal line plots in the centre of SiN windows at red lines in (A–C). (G) The bar graph of output currents of 5–20 W-thickness, which is calculated by subtracting the Si frame average current from the SiN window current. Signal is slightly reduced in accordance with thin film becomes thick. Each bar of output current was averaged from five SiN holders. (H) PSNR of 5–20 nm W-thickness on SiN film. Scale bar, 5 μm in (A).
(TIF)

S2 Fig Electron penetration to the W-coated SiN film by various EB accelerations. (A) Analysis of the electron trajectory and penetration in W-coated SiN film and water using a Monte Carlo (MC) simulation. MC simulation analysis of W-coated SiN film, using CASINO ver. 2.42 [18]; the electron trajectory area in a 10-nm W-layer on 50-nm SiN film is shown. The respective densities of the W-layer and SiN film were 19.3 g/cm^3^ and 3.12 g/cm^3^, and the respective thicknesses were 10 nm and 50 nm. The simulation parameters were set at 1,000,000 electrons, 4 kV EB accelerating voltage, and 30 nm EB spot diameter. (B) An electron trajectory area by MC simulation under 6 kV EB acceleration. (C) Penetrated electrons in water at 3–7 kV EB accelerations, which is calculated at each water depth from the SiN film in a MC simulation. The penetration depth of water was gradually decreasing by reducing EB acceleration. Scale bars, 50 nm in (A) and 100 nm in (B).
(TIF)
