# Supplementary figures and images for: Direct observation of unstained biological samples in water using newly developed impedance scanning electron microscopy
Source: PLoS One. 2019 Aug 20;14(8):e0221296. doi: 10.1371/journal.pone.0221296 (PMC6701803; doi:10.1371/journal.pone.0221296)

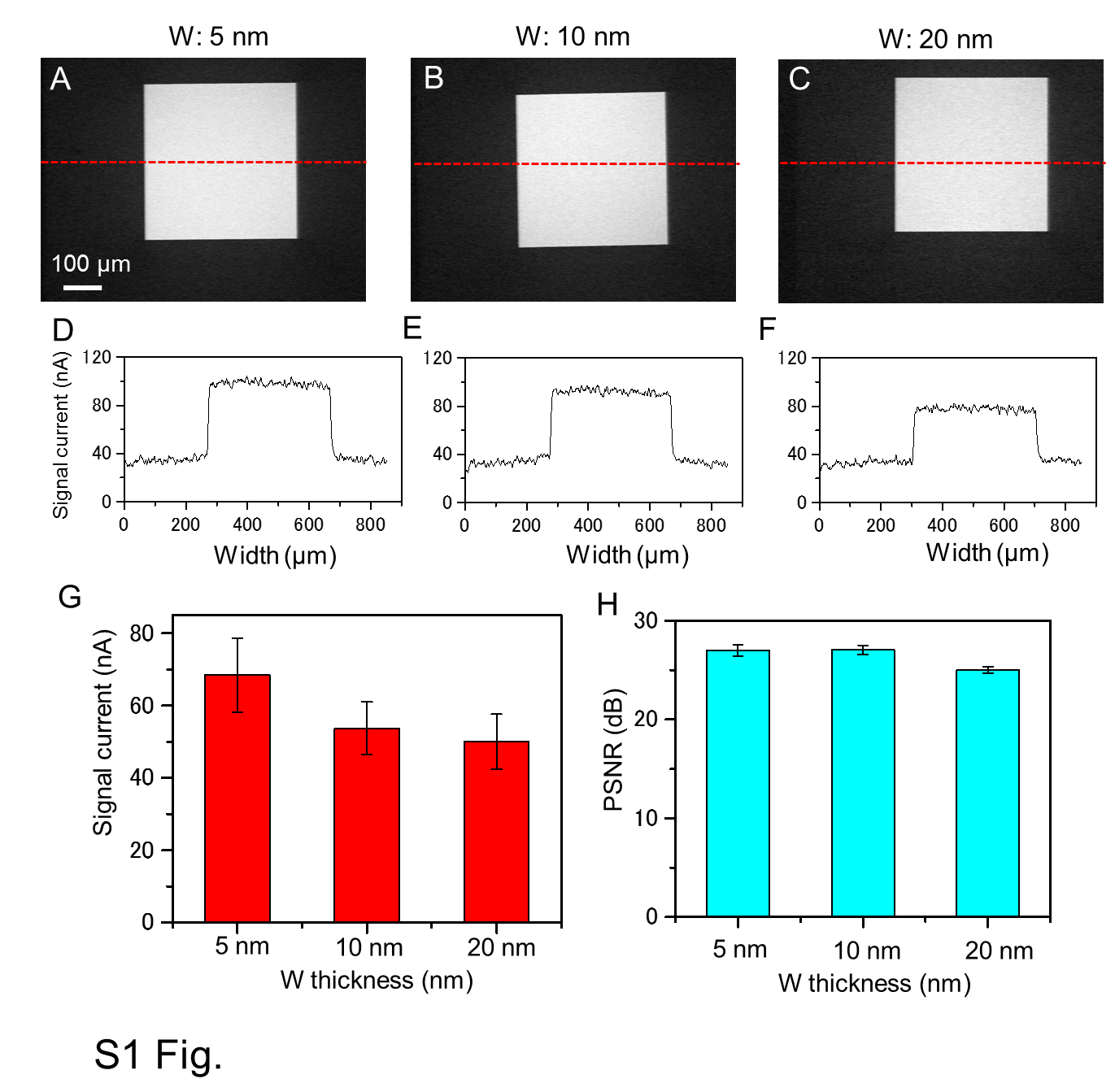

Supplement: S1 Fig — (A)–(C) The current amplitude images of 5–20 nm W-coated SiN film at the atmospheric condition in the holder. (D)–(F) The horizontal line plots in the centre of SiN windows at red lines in (A–C). (G) The bar graph of output currents of 5–20 W-thickness, which is calculated by subtracting the Si frame average current from the SiN window current. Signal is slightly reduced in accordance with thin film becomes thick. Each bar of output current was averaged from five SiN holders. (H) PSNR of 5–20 nm W-thickness on SiN film. Scale bar, 5 μm in (A). (TIF) [file pone.0221296.s001.tif]

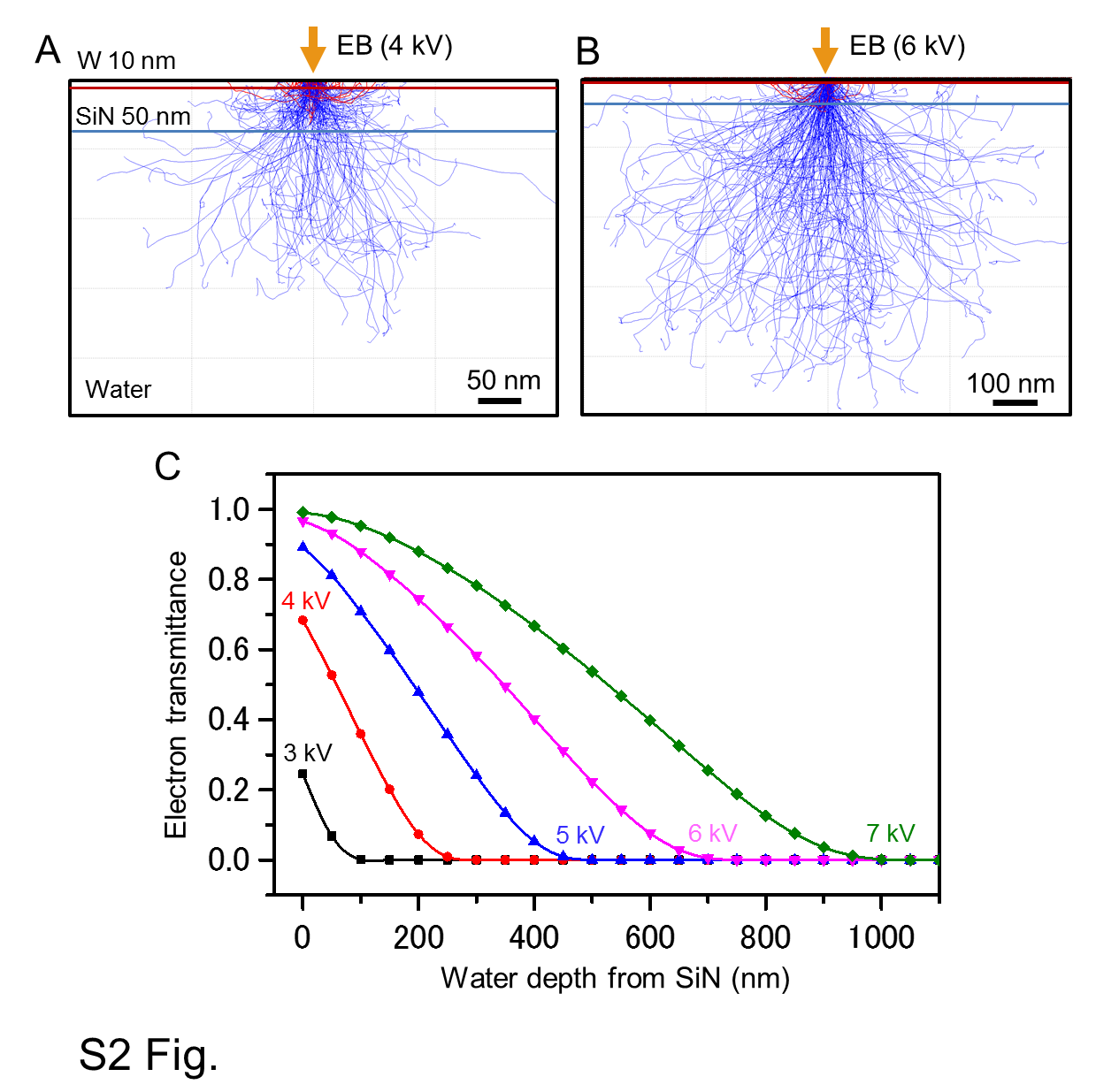

Supplement: S2 Fig — (A) Analysis of the electron trajectory and penetration in W-coated SiN film and water using a Monte Carlo (MC) simulation. MC simulation analysis of W-coated SiN film, using CASINO ver. 2.42 [18]; the electron trajectory area in a 10-nm W-layer on 50-nm SiN film is shown. The respective densities of the W-layer and SiN film were 19.3 g/cm3 and 3.12 g/cm3, and the respective thicknesses were 10 nm and 50 nm. The simulation parameters were set at 1,000,000 electrons, 4 kV EB accelerating voltage, and 30 nm EB spot diameter. (B) An electron trajectory area by MC simulation under 6 kV EB acceleration. (C) Penetrated electrons in water at 3–7 kV EB accelerations, which is calculated at each water depth from the SiN film in a MC simulation. The penetration depth of water was gradually decreasing by reducing EB acceleration. Scale bars, 50 nm in (A) and 100 nm in (B). (TIF) [file pone.0221296.s002.tif]
